# Location of the Mandibular Canal and Thickness of the Occlusal Cortical Bone at Dental Implant Sites in the Lower Second Premolar and First Molar

**DOI:** 10.1155/2013/608570

**Published:** 2013-11-03

**Authors:** Jui-Ting Hsu, Heng-Li Huang, Lih-Jyh Fuh, Rou-Wei Li, Jay Wu, Ming-Tzu Tsai, Yen-Wen Shen, Ming-Gene Tu

**Affiliations:** ^1^School of Dentistry, College of Medicine, China Medical University, 91 Hsueh-Shih Road, Taichung 40402, Taiwan; ^2^Department of Dentistry, China Medical University and Hospital, Taichung 404, Taiwan; ^3^Department of Biomedical Imaging and Radiological Science, China Medical University, Taichung 404, Taiwan; ^4^Department of Biomedical Engineering, Hungkuang University, Taichung 433, Taiwan

## Abstract

The objective of this study was to evaluate the location of the mandibular canal and the thickness of the occlusal cortical bone at dental implant sites in the lower second premolar and lower first molar by using dental cone-beam computed tomography (CBCT). Seventy-nine sites (47 second premolar and 32 first molar sites) were identified in the dental CBCT examinations of 47 patients. In this study, 4 parameters were measured: (1) MC—the distance from the mandibular canal to the upper border of the mandible; (2) CD—the distance from the mandibular canal to the buccal border of the mandible; (3) MD—the distance from the mandibular canal to the lingual border of the mandible; (4) TC—the thickness of the cortical bone at the occlusal side. A statistical analysis was employed to compare the size and differences between these 4 parameters at the lower second premolar and lower first molar. Regarding the MC and MD, the experimental results showed no statistical difference between the first molar and second premolar. However, the TC for the second premolar was greater than that of the first molar. Thus, careful consideration is necessary in choosing the size of and operation type for dental implants.

## 1. Introduction

The location of the mandibular canal is a critical factor that can influence dental implant surgery [1–5]. Dental implant surgery demonstrates a 6.5%–37% incidence of temporary or permanent paralysis, or even sensory loss, because of inferior alveolar nerve damage in the mandibular canal resulting from the poor assessment of bone length and the subsequent use of implant bodies of excessive lengths [2, 3, 6–8]. Therefore, to avoid damage, it is crucial to properly assess the mandibular canal location in the mandible before dental implant procedures. In addition to dental implant surgery, the inferior alveolar nerve may also be damaged by osteotomies or fracture repair; thus, a strong understanding of the intrabony anatomy of the mandibular canal is required before conducting dental implant surgery or operative procedures (e.g., sagittal split osteotomies or placement of cortical fixation screws). Furthermore, the cortical bone thickness of the alveolar bone at the implant site is a critical factor affecting the success of dental implant surgery, because the primary stability of the implant body insertion in the alveolar bone increases with the thickness of the cortical bone [9–13]. Superior osseointegration enhances the long-term survival rate of the implant body.

Although the location of the mandibular canal in the mandible can be precisely determined by conducting biopsies on cadaveric mandibles [1, 14, 15], this method is inapplicable to clinical surgery. Prior to dental implant procedures, dentists currently use panoramic radiography to assess the location of the mandibular canal in the mandible [5, 16, 17]; however, distortion of 2D panoramic radiography often results in miscalculation of the mandibular canal location [14, 18]. Therefore, cross-sectional images are crucial references when assessing the location of the mandibular canal before dental implant procedures [18, 19]. Although cross-sectional images can be obtained using conventional tomographic imaging [15, 16, 18], their accuracy is inferior to that of 3D computed tomography (CT) when measuring the location of the mandibular canal [16, 19]. Similarly, CT can accurately measure the cortical bone thickness of the alveolar bone [20, 21]. Nevertheless, because CTs require high doses of radiation, the technique is not recommended for dental implant procedures unless more than 8 implants are required [17]. Recently, dental cone-beam computed tomography (dental CBCT), which requires a lower radiation dose, has been frequently used in dental diagnosis, treatment, and research [16, 22–27]. In addition to employing lower doses of radiation, dental CBCT possesses greater spatial resolution than CT, making it an ideal preoperative assessment tool for dental implant surgery [16, 28].

The relative location of the mandibular canal in the mandible is information that is indispensable to clinicians before conducting dental implant surgery. However, few studies have focused on using CT to measure the location of the mandibular canal in the mandible [4]. In addition, the thickness of the cortical bone at the implant site is a critical factor affecting the survival rate of the implant body. In this study, we used dental CBCT to locate the mandibular canal and measure occlusal cortical bone thickness at dental implant sites in the lower second premolar and lower first molar.

## 2. Materials and Methods

### 2.1. Dental CBCT Examinations of Patients

Dental CBCT images were collected from 47 patients (aged 52 ± 12 years (mean ± SD), range 28–83 years, 26 males and 21 females). The patients were healthy and either fully lower edentate or missing a lower second premolar or first molar. Seventy-nine sites were identified in the dental CBCT examinations of 47 patients: 47 second premolar and 32 first molar sites. The dental CBCT (i-CAT, Imaging Sciences International, Hatfield, PA, USA) scans were performed at the following technical parameters: 120 kVp, 47 mA, 20 s scanning time, 250 *μ*m voxel resolution, and 160 × 127.75 mm field of view (diameter × high).

### 2.2. Measurement of the Mandibular Canal Position and the Thickness of the Occlusal Cortical Bone

**Figure 1:**
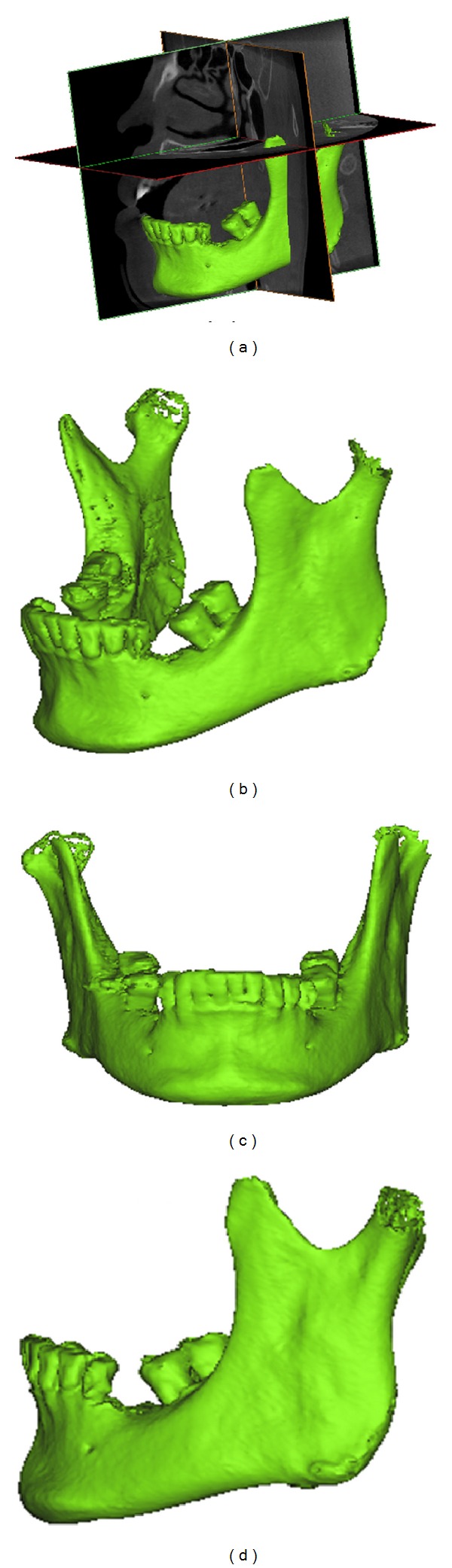
(a) The 3 orthogonal sections of the dental CBCT; (b) 3D model of the mandibular bone from the same angle as (a); (c) frontal view of the mandible rotated to a horizontal plane and parallel to the occlusal plane; (d) side view of the mandible rotated to a horizontal plane and parallel to the occlusal plane.

**Figure 2:**
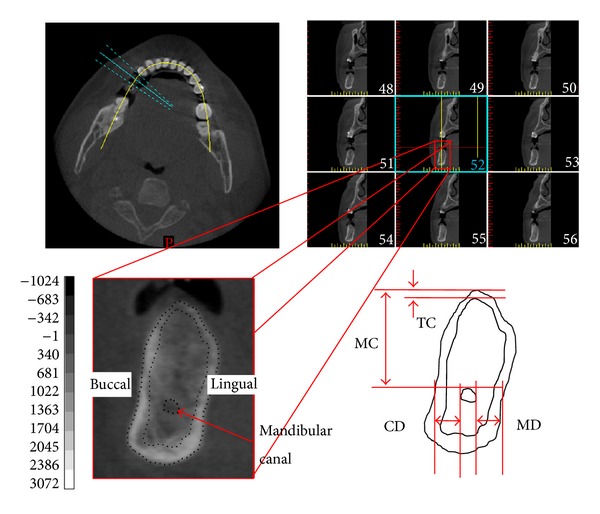
Upper left: axial slice of the mandible with planning and orthoradial lines along the mandibular arch. Upper right: multiple orthoradial reconstructions corresponding to the orthoradial lines visible on the axial slice. Lower half: measurement of the location of the mandibular canal and the thickness of the occlusal cortical bone on the selected slice (second premolar). MC: the distance from the mandibular canal to the upper border of the mandible; CD: the distance from the mandibular canal to the buccal border of the mandible; MD: the distance from the mandibular canal to the lingual border of the mandible; TC: the thickness of the cortical bone at the occlusal side.

Before measuring the positions of the mandibular canal and thickness of the occlusal cortical bone, a coordinate system was created for each mandibular bone by using medical imaging software (Mimics, Materialise, Leuven, Belgium) and the “reslice” function. The mandible of each patient was rotated with the occlusal plane parallel to the horizontal plane and then further rotated with the mandible centered on the midsagittal plane of the image and perpendicular to the occlusal plane (Figure 1). Subsequently, continual buccolingual (cross-sectional) images of the mandibular bone were created using the “online reslice” function of Mimics. The central buccolingual image of the missing tooth (the lower second premolar or lower first molar) was selected to measure the position of the mandibular canal and the thickness of the occlusal cortical bone. In this study, 3 parameters of the mandibular canal location were measured: (1) MC—the distance from the mandibular canal to the upper border of the mandible; (2) CD—the distance from the mandibular canal to the buccal border of the mandible; (3) MD—the distance from the mandibular canal to the lingual border of the mandible. In addition, one parameter for occlusal cortical bone thickness was measured: TC—the thickness of the cortical bone at the occlusal side (Figure 2). Refer to [1, 14, 29]; the 4 length parameters were measured by the observer and examiner error could be neglected based on the statistical analysis.

### 2.3. Statistical Analysis

The measurement accuracy was validated before analyzing the 4 length parameters (MC, MD, CD, and TC). The intraclass correlation coefficient (ICC) was used to determine the reliability of the intraexaminer and interexaminer measurements. Ten buccolingual CBCT slices of the 79 sites (47 second premolar and 32 first molar sites) were randomly selected for evaluating the intraexaminer and interexaminer errors. To calculate the interexaminer error, the 4 parameters of a certain CBCT slice were measured once each by 2 examiners; the ICC values ranged from 0.823 to 0.934. To calculate the intraexaminer error, the 4 parameters of a certain CBCT slice were measured 5 times by a single examiner; the ICC values ranged from 0.893 to 0.975. These values indicate that the intraexaminer and interexaminer error of this method could be neglected in this study.

The mean and standard deviation and coefficient of variation (CV) were calculated for all measurements. The Shapiro-Wilk test was used to determine if the measurements conformed to a normal distribution. The two-sample *t*-test was used to compare the differences in TC, MC, MD, and CD between the second premolar and the first molar. All statistical analyses were performed using OriginPro software (version 8, OriginLab, Northampton, MA, USA). The level of statistical significance was set at *P* < .05.

## 3. Results 

Table 1 lists summarized measurements of the 4 length parameters in the 2 groups. The experimental data were normally distributed (*P* < .05). For the 47 cases of absent second premolars, the TC, MC, MD, and CD were 2.38 ± 0.49 (mean ± SD) mm, 15.88 ± 3.41 mm, 3.93 ± 1.05 mm, and 4.08 ± 0.98 mm, respectively. For the 32 cases with absent first molars, the TC, MC, MD, and CD were 1.72 ± 0.39 mm, 16.15 ± 2.71 mm, 4.00 ± 0.90 mm, and 4.72 ± 1.27 mm, respectively. In addition, excluding the TC and CD, the CVs for the second premolars were larger than those of the first molars.

**Figure 3:**
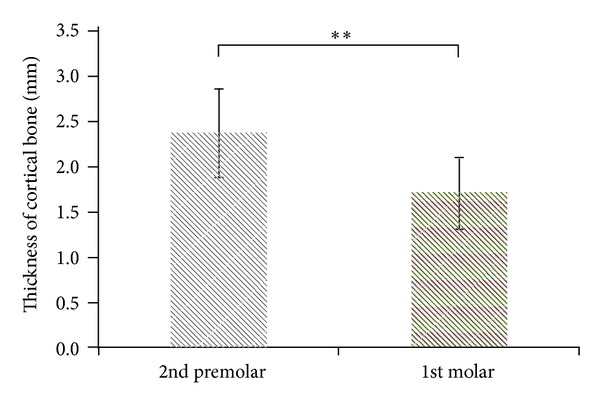
The TC (thickness of the cortical bone at the occlusal side) at the second premolar and first molar position (**P < .001).

**Figure 4:**
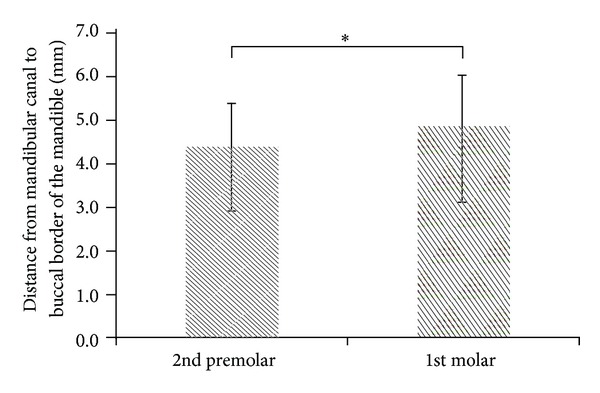
The CD (distance from the mandibular canal to the buccal border of the mandible) at the second premolar and first molar position (*P < .05).

**Figure 5:**
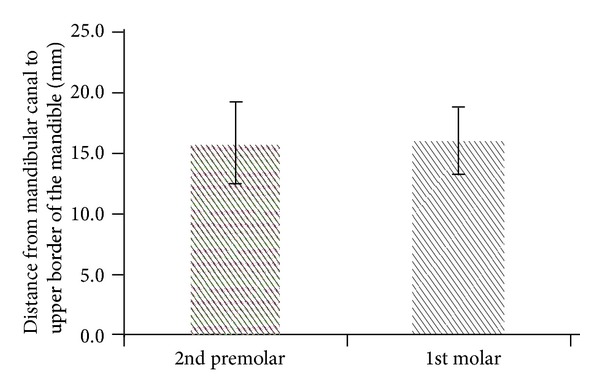
The MC (distance from the mandibular canal to the upper border of the mandible) at the second premolar and first molar position (*P* > .05).

**Figure 6:**
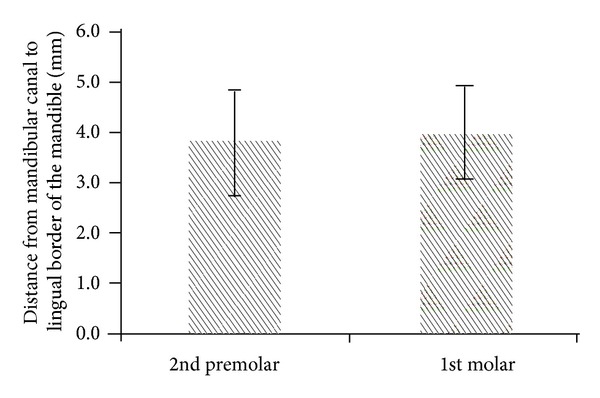
The MD (distance from the mandibular canal to the lingual border of the mandible) at the second premolar and first molar position (*P* > .05).

Comparing the differences among the 4 parameters at the second premolar and first molar, the TC of the second premolar was greater than that of the first molar (*P* < .001; Figure 3), and the CD of the second premolar was less than that of the first molar (*P* < .05; Figure 4). The difference between the second premolar and first molar for the MC and MD was not statistically significant (*P* > .05; Figures 5 and 6).

## 4. Discussion

Dental implant surgery has been popularized in recent years. However, inferior alveolar nerve damage in the mandibular canal can cause postoperative paralysis in the mandible when implant bodies are excessively long or too deeply inserted. In addition, the implant body can loosen when poor quality and quantity of the host bone causes instability. Although previous studies have measured the mandibular incisive canal using dental CBCT [30], no studies have employed this method to measure posterior and mandibular canal locations, or the thickness of the cortical bone. In the current study, we used dental CBCT to measure the relative location of the mandibular canal in the alveolar bone and the thickness of the cortical bone in Asian patients with absent first molars or second premolars. This information can serve as a reference for dentists in determining the optimal implant body lengths or surgical approaches before conducting implants.

The most direct method for measuring the location of the mandibular canal is to measure cadaveric mandibles. Serhal et al. [18] recruited 18 fully or partially edentulous patients, employing a digital sliding caliper to measure the distance from the alveolar crest to the mental foramen. The results were used to compare the differences in the measurements derived through panoramic radiographs, spiral tomograms, and CT scans. The experimental results indicated that the deviation of panoramic radiography was significantly greater than that of the spiral tomograms or CT. It is recommended that cross-sectional imaging be used in the preoperative planning of dental implants. Kaya et al. [5] and Jacobs et al. [29] asserted that spiral CT facilitated accurate measurements of the anterior loop of the mental nerve and mandibular incisive canal. Moreover, Yang et al. [14] used calipers to measure the superior bone height of the inferior alveolar canals in 4 edentulous human cadaver mandibles and compared the differences for measurements using 3D spiral CT. The experimental results indicated that CT could be used to accurately measure the inferior alveolar canal. Dental CBCT was used in the current study because it possesses greater spatial resolution and uses a lower radiation dose than CT or spiral tomograms [16, 31].

Previous studies have attested that dental CBCT provides high accuracy levels for measuring length [32–34]. Therefore, no phantoms or dry skulls were used in this study to verify measurement accuracy. Although grayscale and Hounsfield unit values can be used to determine tissue type in CT or dental CBCT images, this study employed observer measurements for the 4 length parameters. This approach was similar to that of previous studies, which used CT to measure the mandibular canal, mandibular incisive canal, or inferior alveolar canal [1, 14, 29]. The executed statistical tests indicated that the measurement results were not affected by intraexaminer or interexaminer error.

In previous studies measuring the location of the mandibular canal in the mandible, Levine et al. [4] used CT to measure the mandibles of 50 patients. The experimental measurement results showed a 17.4 ± 3.0 mm length from the mandibular canal of the first molar to the alveolar crest, which was slightly longer than the 16.15 ± 2.71 mm measured in this study. The patients in the work of Levine et al. [4] did not have missing teeth, which differed from the recruitment of patients with missing teeth in the present study. Furthermore, Levine et al. [4] measured the distance between the mandibular canal and alveolar crest, which should theoretically be less than the distance from the mandibular canal to the upper border of the mandible (MC); however, the experimental results of Levine et al. [4] (17.4 ± 3.0 mm) were slightly longer than those of the current study (16.15 ± 2.71 mm). This difference could be caused by the various races of patients used in each study. The patients in the current study specifically were all of Asian ethnicities, which typically possess smaller mandibles compared to participants in the work of Levine et al. [4] (who were presumably all Americans). This finding is further verified because the distance from the mandibular canal to the lingual border of the mandible (MD) in the work of Levine et al. [4] (4.9 + 1.3 mm) was also greater than that of the current study (4.00 ± 0.90 mm). Serhal et al. [1] used a digital sliding caliper to measure the mandibular canal location in 6 fresh human cadaveric mandibles, dividing the mandible into 3 sections and measuring the distance from the upper border of the alveolar crest to the upper border of the mandibular canal, which was the same MC measurement used in the current study. The experimental results indicated that the MCs of the 3 sections were 13.53 ± 4.96 mm, 11.92 ± 4.05 mm, and 11.47 ± 4.36 mm. These values were less than the 16.15 ± 2.71 mm obtained in the current study, primarily because 4 of the 6 mandibles used in Serhal et al. [1] were completely edentulous. Complete, long-term edentulism results in alveolar bone resorption, reducing the MC.

The experimental results of the current study showed no statistical difference between the MC for the lower second premolar (15.88 ± 3.41 mm) and lower first molar (16.15 ± 2.71 mm), indicating that implant bodies with similar lengths can be used at both sites. In addition, when planning dental implants, it may be safer to underestimate (rather than overestimate) the distance to the mandibular canal [1]. Typically, 1-2 mm of safety space is retained in clinical practice [35]. Based on the experimental results (mean MC of both groups), the 13 mm dental implant can be selected for both groups. However, the measurement results of this study also indicated that the lowest MC was only 10.56 mm and 11.25 mm for the lower second premolar and lower first molar, respectively. Consequently, in future dental implant procedures, we suggest that clinicians use dental CBCT to confirm the optimal length of the implant body. In addition, when dental implants of identical length are placed, the initial stability of the dental implant and stress and strain distribution to the surrounding bone remains distinct for the second premolar and first molar. Moreover, the experimental results showed that the CD at the lower second premolar (4.08 ± 0.98 mm) was smaller than that of the lower first molar (4.72 ± 1.27 mm). Thus, when monocortical bone plates are necessary for fixing osteotomies, screws should be carefully selected to prevent unnecessarily long or thick screws from causing inferior alveolar nerve injuries.

The majority of previous studies that have examined cortical bone thickness in the mandible have measured buccal-side cortical bone thickness [36, 37]. These measurements have been employed as a reference for miniscrew insertion on the buccal side when assessing orthodontic treatments. Few studies have measured cortical bone thickness at the site of the missing teeth, but numerous studies have used CT to measure the cancellous bone density at the tooth implant site [38–40]. These measurements primarily adopt the directly proportional relationship between the Hounsfield unit (HU) and bone density, which is the so-called bone density in HU [38–40] or radiographic bone density [41, 42]. Scant studies have used CT or dental CBCT to measure the thickness of the cortical bone at the site of the missing teeth. Sato et al. [20] measured the cortical bone thickness of the mandible at the lower first molar and lower second molar in various directions in 27 Japanese skulls. However, the samples employed in Sato et al.'s study [20] were not edentulous, which is different from the approach adopted in the present study (i.e., the occlusal-side cortical bone thickness was only measured at the site of a missing tooth). Nevertheless, the experimental results in the study by Sato et al. indicated that the thickness of the cortical bone at the first molar of the mandible was 1.2–2.8 mm, whereas that adjacent to the lingual side of the alveolar crest was 1.6 mm [20], and the current study achieved a similar measurement (1.72 mm). Miyamoto et al. [43] used CT to measure the cortical bone thickness of the alveolar bone at 127 missing teeth sites in 31 mandibles. The experimental results suggested that the thickness of the occlusal cortical bone of the mandible at the site of the missing teeth was 2.22 ± 0.47 mm (range 0.79–3.21); however, Miyamoto et al. [43] did not examine the locations of various teeth. In the current study, the average thickness of the cortical bone at the lower second premolar and first molar was 2.38 mm and 1.72 mm, respectively; these 2 values were, respectively, higher and lower than the 2.22 mm proposed by Miyamoto et al. [43].

Regarding measurements in the current study, the thickness of the occlusal-side cortical bone of the lower first molar (1.72 ± 0.39 mm) was less than that of the lower second premolar (2.38 ± 0.49 mm). An increasingly thick cortical bone provides stronger primary stability for an implant body [9–13]. Consequently, the primary stability of the implant body for the lower second premolar may be superior to that of the lower first molar. A thick cortical bone reduces the bone strain surrounding the implant body, thereby decreasing the probability of marginal bone loss [12, 44]. Therefore, in addition to understanding cancellous bone density before dental implant surgery, cortical bone thickness can also be used as a reference for determining whether to adopt the single-stage surgical approach (immediate occlusal force) or the 2-stage surgical approach (3–6 mo of osseointegration before occlusal force) in future dental implant surgery.

This study was subject to some limitations. First, we compared only the location of the mandibular canal in the mandible at the first molar and second premolar and the thickness of the occlusal cortical bone and did not evaluate the locations or sites of other missing teeth. Second, we selected only 3 spatial length parameters to measure the location of the mandibular canal in the mandible, neglecting the thickness of the mandibular canal. Third, the effects of sex and age were not investigated because the sample size was insufficient.

## 5. Conclusion

We used dental CBCT to measure the location of the mandibular canal in the mandible and the thickness of the occlusal cortical bone in patients with an absent first molar or second premolar. Based on the 47 evaluated patients, the results showed no statistical difference between the MC for the first molar and second premolar. However, the TC for the second premolar was greater than that of the first molar. Therefore, the primary stability of the implant body of the first molar may be lower than that of the second premolar, which should be carefully considered during dental implant surgery. Moreover, further thought should be given to the time of occlusal loading initiation in the future.

## Figures and Tables

**Table 1:** Measurements of TC, MC, MD, and CD at the second premolar and first molar.

		Mean (mm)		SD (mm)	CV (%)	Max (mm)	Min (mm)
Lower second premolar	TC	2.38	±	0.49	20.49	3.42	1.60
	MC	15.88	±	3.41	21.50	23.75	10.56
	MD	3.92	±	1.05	26.76	6.34	1.65
	CD	4.08	±	0.98	24.90	6.43	2.63

Lower first molar	TC	1.72	±	0.39	22.62	2.60	1.00
	MC	16.15	±	2.71	16.75	23.64	11.25
	MD	4.00	±	0.90	22.46	5.52	1.86
	CD	4.72	±	1.27	26.82	8.52	2.56

SD: standard deviation; CV: coefficient of variation (100 × SD/mean); MC: the distance from the mandibular canal to the upper border of the mandible; CD: the distance from the mandibular canal to the buccal border of the mandible; MD: the distance from the mandibular canal to the lingual border of the mandible; TC: the thickness of the cortical bone at the occlusal side.

All variables were normally distributed (Shapiro-Wilk test, *P* > .05).
